# Histological and Genetic Diversity in Ovarian Mucinous Carcinomas: A Pilot Study

**DOI:** 10.3390/curroncol30040307

**Published:** 2023-04-04

**Authors:** Sultana Razia, Kentaro Nakayama, Hitomi Yamashita, Tomoka Ishibashi, Masako Ishikawa, Kosuke Kanno, Seiya Sato, Satoru Kyo

**Affiliations:** Department of Obstetrics and Gynecology, Shimane University School of Medicine, Izumo 6938501, Japan; raeedahmed@yahoo.com (S.R.); memedasudasu1103@gmail.com (H.Y.); tomoka@med.shimane-u.ac.jp (T.I.); m-ishi@med.shimane-u.ac.jp (M.I.); kanno131039gyne@gmail.com (K.K.); sato_seiya9534@yahoo.co.jp (S.S.); satoruky@med.shimane-u.ac.jp (S.K.)

**Keywords:** histological diversity, genetic diversity, ovarian mucinous carcinoma, ovarian cancer

## Abstract

Tumor heterogeneity remains an ongoing challenge in the field of cancer therapy. Intratumor heterogeneity significantly complicates the diagnosis of cancer and presents challenging clinical problems due to resistance to drug therapy. This study aimed to elucidate the genetic changes histologically (mucinous cystadenoma (MCA), mucinous borderline tumor (MBT), and mucinous ovarian carcinoma (MOC)) in a portion of mucinous ovarian tumors within the same sample. Seven tumor samples obtained from different patients were used to evaluate the genetic mutations in each component. Intratumor genetic heterogeneity was observed in all patients; among them, *BRAF* (V600E) and *p53* (T118I, P142S, T150I, and T170M) point mutations were observed in the MBT component, while *KRAS* (G12D and G13D) and *PIK3CA* (E545K) mutations were found in the MOC component. The current findings suggest that diverse genetic alterations occur in mucinous tumors, according to tumor histology. Tumor heterogeneity and genetic diversity in mucinous ovarian tumors might be the cause of treatment failure. Knowledge of intertumor heterogeneity may lead to an increased understanding of the tumor response to treatment.

## 1. Introduction

Ovarian cancer is the most lethal gynecological malignancy worldwide [1]. In the female population, ovarian cancer accounts for approximately 3.6% of all cancer types [2]. The most common ovarian carcinoma is the serous type. Mucinous histology is uncommon, accounting for approximately 2–10% of all subtypes of epithelial ovarian carcinomas [3,4]. Primary mucinous ovarian carcinomas are distinct from other types of epithelial ovarian cancers in terms of presentation and outcome [5,6,7]. The prognosis of mucinous ovarian carcinoma is favorable when identified at an early stage; however, its prognosis is poor at advanced stages since it has a tendency to chemoresistance, which could be partly explained by the hypothesis that mucinous ovarian carcinomas are genetically stable [8]. The most commonly diagnosed ovarian epithelial borderline tumor in Japan is the mucinous type, whereas the most commonly diagnosed in Western countries is the serous type [9,10,11]. Therefore, understanding the mechanisms contributing to the development and progression of mucinous ovarian cancer, as well as the novel therapeutic approaches, is urgently needed.

Histological heterogeneity with a clear progression from benign to borderline to carcinoma has been frequently observed in mucinous ovarian tumors. These histologic heterogeneities indicate that the progression from benign to carcinoma in ovarian mucinous tumors occurs due to the accumulation of multiple genetic events [9]. The most common genetic alternation of mucinous ovarian tumors occurs in the pathway of mitogen-activated protein kinase (MAPK) (RAS-RAF-MEK-ERK-MAP). *BRAF* and *KRAS* mutations are components of the MAPK cascade. *KRAS* mutations are frequent in mucinous ovarian tumors and occur in 40–50% of patients with mucinous ovarian cancer [12]. The quality sequencing data of 63 patients with primary ovarian mucinous tumors were obtained, including 26 with borderline tumors and 37 with carcinomas. Deleterious somatic mutations were observed in 13 genes: *KRAS*, *TP53*, *CDKN2A*, *PIK3CA*, *PTEN*, *BRAF*, *FGFR2*, *STK11*, *CTNNB1*, *SRC*, *SMAD4*, *GNA11*, and *ERBB2*. *KRAS* mutations remain the most frequently observed alterations in patients with carcinomas (64.9%) and mucinous borderline tumors (92.3%) [13]. *TP53* mutations occur more frequently in carcinomas than in borderline tumors (56.8% and 11.5%, respectively) [13]. We previously performed a direct sequence analysis on 38 tumor specimens, including 16 mucinous ovarian carcinoma (MOC), 10 mucinous borderline tumor (MBT), and 12 mucinous cystadenoma (MCA) specimens, to elucidate the genetic profile of mucinous tumors; we found that *BRAF* mutations were more common in MBT than in MOC. *KRAS* mutations were relatively high (43.8%) in MOC but were relatively low (20%) in MBT. None of the mucinous specimens showed *TP53* mutations; *PIK3CA* mutations were only detected in one MCA specimen [14]. However, mucinous cancers appear to have intratumoral genetic heterogeneity, which poses an intriguing challenge for molecular profiling and the creation of potential customized therapy approaches. Furthermore, only limited research has looked into the genetic changes in mucinous ovarian cancers.

Although several studies have reported the genetic alternation of mucinous ovarian carcinomas [12,13], genetic alternation in a tumor with the coexistence of benign, borderline, and malignant components is not frequently observed. In the current study, we aimed to elucidate the genetic heterogeneity of mucinous ovarian carcinomas according to tumor histology in Japanese patients. Seven tumor specimens from different patients that encompassed the MCA, MBA, and MOC components were used to evaluate the genetic alterations in each component. However, to our knowledge, no existing studies in Japan have compared seven different tumor samples that contain each of the three (MCA, MBA, and MOC) components.

## 2. Materials and Methods

### 2.1. Tumor Samples

Seven ovarian mucinous tumor specimens were obtained from different patients admitted to the Department of Obstetrics and Gynecology, Shimane University Hospital (Izumo, Japan). The MCA, MBA, and MOC components of each tumor were removed and subjected to mutation analysis. The histological diagnoses of MCA, MBA, and MOC components were made based on the results of conventional histopathological examination of sections stained with hematoxylin and eosin (H&E). The categorization of the tumors was performed according to the criteria of the World Health Organization. The International Federation of Gynecology and Obstetrics classification system was followed for the staging of tumors. All patients underwent total abdominal hysterectomy, bilateral salpingo-oophorectomy, and omentectomy with or without pelvic and para-aortic lymph node dissection. All patients received platinum and taxane-based combination chemotherapy. An expert in gynecological pathologist (N.I.) reviewed all resected specimens from each patient for diagnosis of the disease. The acquisition of tumor tissue was approved by the institutional review board of Shimane University (approval no. 2004-0381, 5 March 2007).

### 2.2. Microdissection and Deoxyribonucleic Acid Extraction

Formalin-fixed, paraffin-embedded tissues (FFPE) of each portion (MCA, MBA, and MOC) were used for the extraction of Deoxyribonucleic acid (DNA). A gynecologic oncologist (K.N.) and pathology technician (K.I.) carefully selected the FFPE blocks of mucinous ovarian tumors, and identified the portions of MCA, MBT, and MOC appropriate for microdissection. FFPE blocks were serially sectioned to a thickness of 10 mm. After being deparaffinized with xylene and hydrated using graded alcohol, the microdissected tissue samples were incubated with ATL buffer and proteinase K for overnight at 56 °C. The genomic DNA for mutation analysis was extracted from the microdissected samples using a QIAmp DNA Micro Kit (Qiagen, Valencia, CA, USA), according to the manufacturer’s instructions.

### 2.3. Direct Sequence Analysis

Extracted genomic DNA obtained from microdissected formalin-fixed paraffin-embedded tissues of each component (MCA, MBA, and MOC) was used to amplify by polymerase chain reaction (PCR) using different primers. The primers used for amplification were *KRAS*, *BRAF*, *PIK3CA*, and *TP53*. This study focused on exons that are reported to harbor most mutation in each gene. The sequence of the primer and the thermal cycle profile used for all gene amplification have been described previously [15].

All products that were amplified by PCR were sequenced at Beckman Counter (Danvers, MA, USA) and analyzed using the Mutation Surveyor DNA Variant Software (Tokyo, Japan).

Using the Catalogue of Somatic Mutations in Cancer, the pathogenicity of each mutation was evaluated [16].

## 3. Results

**Figure 1:**
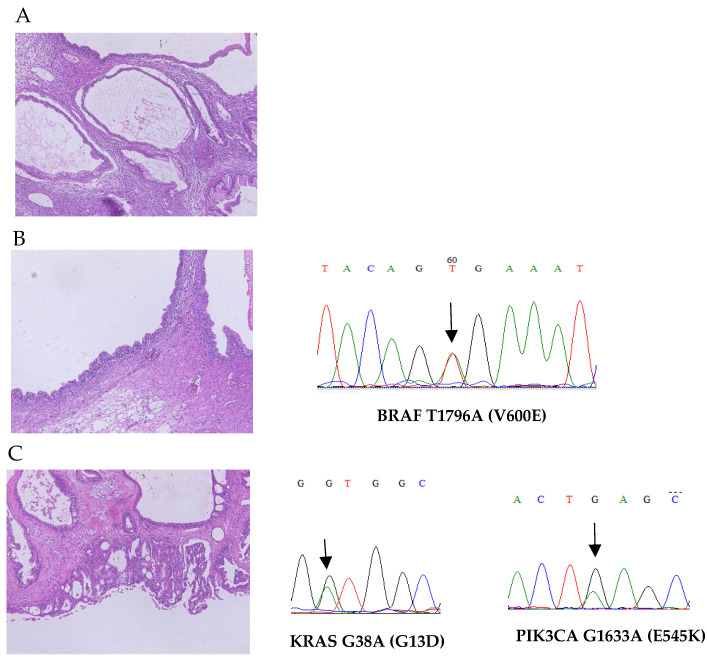
Heterogenous mutation pattern within a tumor according to heterogenous tumor histology (Case 1). (**A**) Mucinous cystadenoma showing no mutation. (**B**) Mucinous borderline tumor showing V600E mutation (1796 T > A) in *BRAF.* (**C**) Mucinous ovarian carcinoma showing E545K mutation (1633 G > A) in *PIK3CA* and G13D mutation (38 G > A) in *KRAS*.

**Figure 2:**
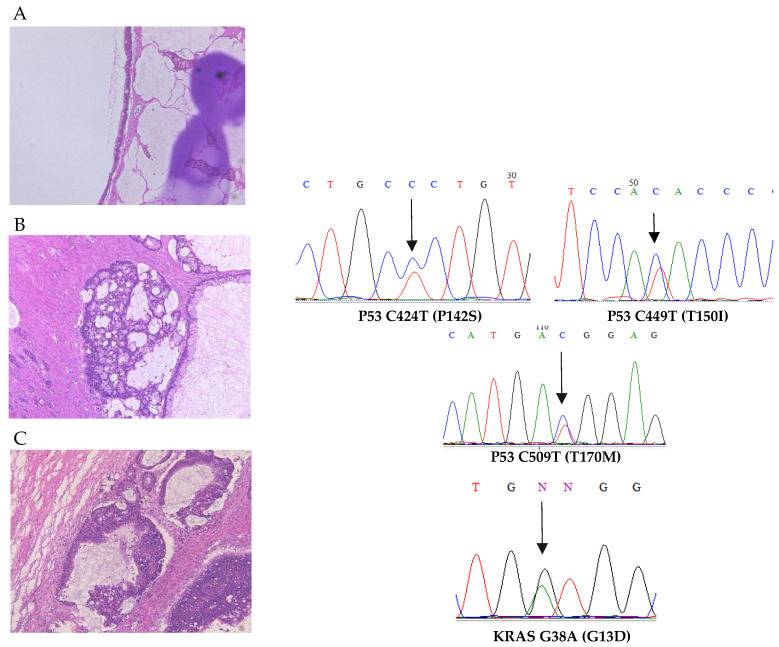
Heterogenous mutation pattern within a tumor according to heterogenous tumor histology (Case 3). (**A**) Mucinous cystadenoma showing no mutation. (**B**) Mucinous borderline tumor showing P142S (424 C > T), T150I (449 C > T), and T170M (509 C > T) mutations in *p53.* (**C**) Mucinous ovarian carcinoma showing G13D mutation (38 G > A) in *KRAS*.

To assess the genetic alterations in mucinous ovarian tumors, direct sequence analysis of seven tumor specimens was performed, and each specimen contained MCA, MBA, and MOC components. Some sequence results from the MCA portion were used in our previous study [15]. The clinical characteristics of the patients are summarized in Table 1. The mean patient age was 62.8 (range, 47–81) years, and the average tumor size was 20 (range, 16–33) cm in diameter. The MCA, MBA, and MOC components were used to assess for *KRAS, BRAF, TP53,* and *PIK3CA* mutations. Intratumoral genetic heterogeneity was observed in all cases. In Case no. 1, *BRAF* (V600E) point mutation was detected only in the MBT component, whereas *KRAS* and *PIK3CA* mutations simultaneously occurred in the MOC component in exon 2 (G13D) and exon 9 (E545K). In the other two cases (Cases 2 and 3), *p53* mutation was detected in the MBT component in exon 4 (T118I) and exon 5 (P124S, T150I, and T170M), while *KRAS* mutation was observed in exon 2 (G12D and G13D) in the MOC component (Table 1, Figure 1 and Figure 2).

## 4. Discussion 

Mucinous neoplasms of the ovary account for 10–15% of ovarian neoplasms. They could be benign, borderline, or malignant. Briefly, mutations in *KRAS* (55%), *CDKN2A* (55%, including deletions), *TP53* (52%), *ARID1A* (10%), *BRAF* (8%), and amplification of *HER2* (28%) are the most frequent abnormalities observed in primary mucinous ovarian cancer [13,17,18,19,20,21,22]. Primary ovarian mucinous tumors with mural nodules are very rare. Mural nodules exist in a wide range of types, including anaplastic carcinomas, true sarcomas, and sarcoma-like mural nodules. Older patients tend to develop mucinous tumors with carcinomatous mural nodules, and have a poor prognosis, with a 50% mortality rate despite adjuvant chemotherapy and/or radiotherapy [23]. One study suggested that anaplastic mural nodules could be more likely to result from *KRAS* mutant tumors [24]. Another study observed aberrant *p53* expression in the mural nodules rather than in the mucinous components [25]. Until now, a number of studies have documented the genetic alternation of mucinous ovarian carcinomas in a variety of individuals. In our study, seven cases of tumor specimens, including the MCA, MBA, and MOC components together, were used to assess the genetic heterogeneity according to tumor histology. Anaplastic mural nodules were not observed in any of the cases.

In the present study, the heterogeneity of genetic alterations in histological features in mucinous tumors was evaluated, and mutational variability was observed in different areas of the same tumor. Mandai et al. (1998) found that tumors with *KRAS* mutations showed the same mutational pattern of nucleotide changes in specimens obtained from different areas in each patient [9]. Takeshima et al. (2001) observed a heterogeneous pattern of *KRAS* point mutations in 5 of 10 (50%) patients and a greater incidence of genetic changes occurring in patients with more atypical areas [10]. However, our results suggest that this finding may not be justified. In our study, genetic alterations were observed in various histological portions of mucinous carcinomas. In one study conducted by Takeshima et al. (2000), to observe the correlation between the heterogeneous histological pattern and genetic alterations in mucinous tumors, it was found that the expression of p53, Ki-67, and c-erbB2 increased according to the histological stage and frequency of genetic abnormalities. Immunohistochemistry was conducted with the antibodies of cell-cycle-related proteins, and the expression of labeling indices *p53* and *Ki-67* increased according to the histological characteristics, which were higher in more atypical areas and the highest expression was observed in a patient of adenocarcinoma. Furthermore, *c-erbB2* expression (30%) was positive only for patients with adenocarcinoma and undetectable in benign tumors. [26]. Another report generated the relationship between benign, borderline, invasive low-grade and high-grade mucinous tumors and found that either a *KRAS* or *CDKN2A* event leads to the initiation of benign tumors. Both events are noticeably more common in MBT and may have additional copy number alterations. Grade 1 MOC are more likely to have a *TP53* mutation and have additional copy number changes. Copy number alteration is a major factor contributing to metastatic progression and grade [22]. Taken together, these and the current results suggest that the histological heterogeneity of ovarian mucinous carcinomas interacts with genetic heterogeneity.

The quantification of intertumoral genetic diversity and its relationship with therapy resistance have been explored in recent years. Several studies have concluded that each tumor cell is genetically distinct [27,28,29]. Both Loeb et al. [30] and Sottoriva et al. [31] reported that a high mutation rate facilitates drug resistance. The genomic profiling of 349 individual glands from 15 colorectal tumors was evaluated and observed high intratumoral heterogeneity, and subclone mixing in distant regions [31]. The presence of unexpected mutations within tumors includes the rapid emergence of resistance to chemotherapeutic agents [30]. Cancer types with a low genetic mutation burden are associated with a long-term response to targeted therapy, whereas gene-unstable cancer types have a short-term response to targeted therapy. Resistance to therapy remains a challenging clinical problem and has no definitive treatment. Knowledge of intertumor heterogeneity may lead to an increased understanding of the tumor response to treatment.

In the present study, most borderline tumors contained *TP53* mutations, whereas the carcinoma portion contained *KRAS* mutations. Previous studies examined the role of tumor-suppressor genes in human thyroid tumorigenesis; the results showed that mutations in the p53 gene are associated with poorly differentiated thyroid tumors [32]. *BRAF* is the most commonly mutated gene, and *KRAS* mutations are the second most common genetic alterations in well-differentiated thyroid cancers [33]. Poorly differentiated carcinomas are disorganized under the microscope and tend to grow and spread faster compared with well-differentiated (grade I) carcinomas. Therefore, the borderline portion that contains *TP53* mutations might transform into a poorly differentiated carcinoma if left untreated.

First, the main limitation of our study was the small sample size; it was difficult to draw a clear conclusion about the findings. Therefore, further investigations with larger study populations are required. Second, we assessed the genetic mutations via Sanger sequencing; hence, the types of gene mutations assessed were limited. Further studies with next-generation sequencing are warranted to determine the molecular mechanisms underlying mucinous ovarian carcinoma. Research is currently being conducted in our laboratory to overcome these limitations.

## 5. Conclusions

Diversity in the histological heterogeneity of ovarian mucinous carcinomas was reported in our study. As mucinous ovarian carcinomas are usually large (typically >15 cm in diameter) and frequently include histologically heterogeneous portions of benign, borderline, and carcinomas, this subtype may become more heterogeneous. Higher genetic and histological diversity within a tumor may increase the risk of drug resistance. Tumor histological heterogeneity, together with tumor genetic diversity in mucinous ovarian tumors, may be the cause of treatment failure. Heterogeneity fuels tumor resistance to treatment; therefore, an accurate assessment of tumor heterogeneity may be essential for the development of effective therapies.

## Figures and Tables

**Table 1:** Heterogeneous genetic alterations in mucinous ovarian tumors.

Case	Tumor Size	Age	Stage (FIGO)	Histologic Category	*KRAS*	*BRAF*	*TP53*	*PIK3CA*
1				cystadenoma	WT	WT	WT	WT
	17	56	IV A	borderline tumor	WT	V600E	WT	WT
				carcinoma	G13D	WT	WT	E545K
2				cystadenoma	WT	WT	WT	WT
	17	77	I A	borderline tumor	WT	WT	T118I	NA
				carcinoma	G12D	WT	WT	WT
3				cystadenoma	WT	WT	WT	WT
	18	63	II C	borderline tumor	WT	WT	P142S, T150I, T170M	WT
				carcinoma	G13D	WT	WT	WT
4				cystadenoma	WT	WT	WT	WT
	16	67	IV A	borderline tumor	WT	WT	E62K, E68K	WT
				carcinoma	WT	WT	WT	WT
5				cystadenoma	WT	WT	WT	WT
	22	81	I A	borderline tumor	WT	WT	D57N	WT
				carcinoma	WT	WT	WT	WT
6				cystadenoma	WT	WT	WT	WT
	17	78	I C	borderline tumor	WT	WT	WT	WT
				carcinoma	G12D	WT	WT	WT
7				cystadenoma	WT	WT	WT	WT
	33	47	III A	borderline tumor	WT	WT	WT	WT
				carcinoma	G12D	WT	WT	WT

FIGO, International Federation of Gynecology and Obstetrics.

## Data Availability

The data presented in this study are available from the corresponding author (K.N.) upon reasonable request.
